# Supplementary material for: The Role of Fluorine in F-La/TiO2 Photocatalysts on Photocatalytic Decomposition of Methanol-Water Solution
Source: Materials (Basel). 2019 Sep 5;12(18):2867. doi: 10.3390/ma12182867 (PMC6765986; doi:10.3390/ma12182867)
Supplement: Supplementary file 1 [file materials-12-02867-s001.pdf]

materials

The Role of Fluorine in F-La/TiO_2 Photocatalysts on Photocatalytic Decomposition of Methanol-Water Solution

Miroslava Edelmannová [1,2], Lada Dubnová [3], Martin Reli [1], Vendula Meinhardová [3], Pengwei Huo [4], Urška Lavrenčič Štangar [5], Libor Čapek [3] and Kamila Kočí [1,*]

[1] Institute of Environmental Technology, VŠB-Technical University of Ostrava, 17. listopadu 15/2172, Ostrava 70800, Czech Republic; miroslava.edelmannova@vsb.cz (M.E.); martin.reli@vsb.cz (M.R.); kamila.koci@vsb.cz (K.K.)

[2] Faculty of Materials Science and Technology, VŠB-Technical University of Ostrava, 17. listopadu 15/2172, Ostrava 70800, Czech Republic

[3] Faculty of Chemical Technology, University of Pardubice, Studentská 573, Pardubice 532 10, Czech Republic; lada.dubnova@student.upce,cz (L.D.); vendula.meinhardova@upce.cz (V.M.); libor.capek@upce.cz (L.C.)

[4] School of Chemistry and Chemical Engineering, Jiangsu University, 301 Xuefu Road, Zhenjiang, Jiangsu 212013, China; huopw@ujs.edu.cn

[5] Faculty of Chemistry and Chemical Technology , University of Ljubljana, Večna pot 113, P.O Box 537, 1001 Ljubljana, Slovenia; Urska.Lavrencic.Stangar@fkkt.uni-lj.sl

* Correspondence: kamila.koci@vsb.cz

Supplementary Materials

Characterization of Materials

The specific surface area (S_{BET}) was measured using the Sorptomatic 1990 instrument (Thermo Finnigan, Rodano, Italy). The Brunauer–Emmett–Teller (BET) N_2 physisorption method was determined by employing the Advance Data Processing software according to the BET isotherm.

X-ray powder diffraction (XRD) patterns were obtained using a Rigaku SmartLab diffractometer (Rigaku, Tokyo, Japan) with detector D/teX Ultra 250. The source of X-ray irradiation was Co tube (CoKα, λ_1 = 0.178892 nm, λ_2 = 0.179278 nm) operated at 40 kV and 40 mA. Incident and diffracted beam optics were equipped with 5° Soller slits; incident slits were set up to irradiate area of the sample 10 × 10 mm (automatic divergence slits) constantly. Slits on the diffracted beam were set up to fixed value 14 mm. The powder materials were measured in the reflection mode (Bragg-Brentano geometry). The samples rotated (15 rpm) during the measurement to eliminate preferred orientation effect. The XRD patterns were collected in a 2θ range 5–145° with a step size of 0.01° and speed 0.5 deg.min^{-1}. Measured XRD patterns were evaluated using PDXL 2 software (version 2.4.2.0) and compared with database PDF-2, release 2015. Structural parameters of Anatase were evaluated by Rietveld fitting program MSTRUCT.

Raman spectroscopy was measured using a Nicolet DXR SmartRaman spectrometer (Thermo Fisher Scientific, Waltham, MA, USA) equipped with 780 nm NIR excitation laser. The laser power on the sample was 1 mW, spectra were recorded by collecting of 200 scans and the spectrograph aperture was a 50 μm slit. Raman spectra were recorded in the 55–3500 cm^{-1} wavenumber range.

DRS spectra of the prepared materials were measured in quartz cuvettes by using a GBS CINTRA 303 spectrometer (GBC Scientific Equipment, Victoria, Australia) equipped with integrating sphere. The spectra of catalysts were scanned in the wavelength range 190–900 nm, scan speed 100 nm.min^{-1}, step size 1 nm and slit width of 2 nm. Reflectance was recalculated into the dependence of Kubelka–Munk function (Reli et al., 2015) based on the equation $F(R_\infty) = (1-R_\infty)^2/(2 \times R_\infty)$, where R_∞ is the diffuse reflectance from a semi-infinite layer. This equation were transformed to the dependency $(F(R_\infty)\cdot h\cdot \nu)^{1/2}$ described as $(\alpha\cdot h\cdot \nu)^{1/2}$ against photon energy for determination of band gap energy of indirect semiconductor.

X-ray fluorescence (XRF) was measured on a wave-dispersion X-ray fluorescence spectrometer (Spektroskop MAKC-GV, Spectron NPO Ltd., Sankt-Peterburg Russia) with a top vacuum. Atomic absorption spectroscopy was measured on an Elementar vario EL III instrument (Elementar, Germany).

Chemical components and the binding energies of F-La/TiO_2 were analyzed by XPS (Thermo Scientific ESCALAB 250Xi A1440 system, Waltham, MA, USA).

Electrochemical impedance spectroscopy (EIS) was performed in a 0.5 M Na_2SO_4 solution witha frequency range from 0.1 Hz to 100 kHz at 0.5 V. The amplitude of applied sine wave potential in each case was 5 mV which wascarried out using a ZENNIUM electrochemical workstation (Zah-ner Instruments, Kronach, Germany).

Photoelectrochemical measurements were carried out using a photoelectric spectrometer with a 150 W Xe lamp used as an irradiation source and coupled with the P-IF 1.6 potentiostat (Instytut Fotonowy, Krakov, Poland). The photocurrent responses were recorded using a classical three electrode setup. The platinum wire and Ag/AgCl were used as the counter and reference electrodes, respectively. The working electrode consisted of photocatalyst powder deposited onto indium-tin oxide (ITO) foil coated by polyethylene terephthalate. 0.1 M KNO_3 was used as an electrolyte solution. The photocurrent spectra were recorded within the range of 240–450 nm with the step of 10 nm in the potential range of −0.2 to 1.0 V, step 0.1 V. Before the measurement itself, the measuring cell with electrolyte was purged by argon to ensure an oxygen free environment. The argon purge was also kept constant during the whole measurement.

Photocatalytic Test

The photocatalytic decomposition of methanol-water solution was carried out in a homemade stainless steel batch photoreactor (volume = 348 mL) irradiated with an 8W Hg UVA lamp (λ_{max} = 365 nm), which irradiated solution through quartz glass window on the top of reactor. Each batch was formed by 0.1 g of investigated photocatalyst, 100 mL of methanol solution (12 M) and was mixed to prevent sedimentation of photocatalyst. Before start reaction (illumination), was suspension purged by helium with a constant flow for 45 min. The gaseous samples were analyzed by GC (Shimadzu Tracera GC-2010 Plus) equipped with barrier discharge detector (BID) every hour (in time interval 0–4 h). Each experiment was repeated in order to ensure the reproducibility of the experimental data. The blank tests were also performed.
